# How can the United States make a great stride toward multiracial well-being?

**DOI:** 10.1371/journal.pone.0319320

**Published:** 2025-04-16

**Authors:** Bobby Milstein, Jack Homer, Becky Payne, Paul Reed

**Affiliations:** 1 Rippel Foundation, Morristown, New Jersey, United States of America; 2 Homer Consulting and MIT Research Affiliate, Barrytown, New York, United States of America; 3 US Department of Health and Human Services, Office of Disease Prevention and Health Promotion, Columbia, Washington, United States of America; University of California Los Angeles, David Geffen School of Medicine, UNITED STATES OF AMERICA

## Abstract

In America and around the world, one’s chances for well-being depend on systems that are not yet built for *everyone* to thrive together. Knowing that life expectancy and life evaluation are far below their full potential, with stark injustices by race/ethnicity, we ask: how can the United States make a great stride toward multiracial well-being?. This study explores potential impacts of a federal plan for thriving people and places. We estimate the likely effects of 68 recommendations using ReThink Health’s *Thriving Together Model* (TTM), revised with new data and new features including a multisolving ratio that accounts for greater cost-effectiveness when a proposed action advances multiple goals at once. The TTM is a previously published system dynamics model that simulates changes over time when community assets (both funding and in-kind resources) are invested in four drivers of population well-being (i.e., Vital Conditions, Belonging and Civic Muscle, Fairness in System Design, and Urgent Services Capacity). All drivers work together through a dynamic structure that influences individual states of thriving, suffering, and life expectancy (overall and by race/ethnicity). The model specifies three reinforcing dynamics, including an “expanding the pie” loop that can increase available assets and improve all four well-being drivers over time. Results reveal a plausible scenario over 25 years in which thriving could rise 20 percentage points, suffering could drop 2.5 percentage points, and average life expectancy could grow by 2.6 years – all from equitable progress across racial/ethnic groups. Every subgroup improves, but the greatest gains would likely be among Black and Hispanic Americans. Sensitivity tests confirm that the model’s conclusions are robust across identified uncertainty ranges. The federal plan points the way toward a just transition for multiracial well-being. It does not require new appropriations or authorities: only the will and wherewithal to bring these recommendations to life.

## Introduction

People everywhere aspire to lead long, thriving lives. However, in a diverse, multiracial society fulfilling this desire also hinges on people’s willingness to bridge differences and design systems that assure fair opportunities for everyone to participate, prosper, and reach their full potential [1, 2]. Well-being for individuals (i.e., how they think, feel, and function) is strongly shaped by their surroundings, characterized by a complex system of exposures, opportunities, and choices that each person encounters throughout their lives. In America and around the world, one’s chances for well-being depend on systems that are not yet built for everyone to thrive together. But even systems fraught with entrenched racism and other forms of injustice can be transformed through shared stewardship (i.e., practices for working together around common values) [3, 4].

**DEFINITIONS**

*Well-Being (Thriving, Struggling, Suffering):* Well-being is how people think, feel, and function at an individual and societal level. Individual well-being can be classified into one of three groups based on Gallup’s Life Evaluation Index using the widely-validated Cantril Self-Anchoring Scale [5]. The index classifies people based on responses to two questions about their current and future life evaluation, each on a 0–10 scale:

- *Thriving*: Well-being is strong, consistent, and progressing. Respondents have positive views of their present life situation (7+) and positive views of the next five years (8+)
- *Struggling*: Well-being is moderate or inconsistent. Respondents have moderate views of their present life situation or moderate or negative views of their future
- *Suffering*: Well-being is at high risk. Respondents have poor ratings of their current life situation (4 and below) and negative views of the next five years (4 and below).

*Multiracial Well-Being:* A high prevalence of thriving and low prevalence of suffering across multiple racial/ethnic groups.

Consider, for example, the far-reaching social dynamics of the COVID-19 pandemic, which inflicted much greater harm on the most undervalued racial/ethnic groups across the US, exposed a lack of equitable well-being, [6] and focused attention on the toxic effects of systemic racism [7]. It also propelled new players to join a longstanding movement for well-being and justice. For instance, more than 100 contributors came together shortly after COVID social isolation measures were instituted to produce the *Thriving Together Springboard* [8], which described how interdependent stewards in communities across the country could convert unjust loss into equitable renewal through investments in the vital conditions for health and well-being [9].

That non-governmental effort was followed by a complementary effort across the federal government. In November 2022, following two and half years of deliberations, dozens of departments, agencies, and institutes across the US government (now totaling 47) published a federal interagency plan for Equitable Long-Term Recovery and Resilience (ELTRR) [10]. This plan has been described by the National Academies as a historic, whole-of-government commitment to enhance racial, ethnic, and tribal health equity [11]. The ELTRR was produced by over 150 career professionals, working through both Republican and Democratic administrations, who crafted 78 recommendations to be “integrated and institutionalized into the normal and expected course of policymaking, operations, and funding across federal agencies” [10]. Of the 78 recommendations, 10 address intra-governmental coordination while the other 68 describe specific efforts to improve one or more of the vital conditions and, in many cases, racial/ethnic equity as well.

Evolving from its origins in pandemic recovery, the ELTRR has recently been renamed “*People and Places Thriving: The Federal Plan for Long-Term Resilience”*, reflecting the initiative’s commitment to address thriving in perpetuity [12]. The published recommendations remain unchanged; so, for clarity we use the original term ELTRR throughout this paper when referring to them. In this paper, we ask: to what extent could ELTRR priorities unlock America’s potential for multiracial well-being? To answer that question, we compared three alternative scenarios using a revised version of a previously published simulation model called the *Thriving Together Model* (TTM) [13].

## Materials and methods

### Design of the revised thriving together model

ReThink Health, an initiative of the nonprofit Rippel Foundation, first created the Thriving Together Model in the summer of 2020, with subsequent improvements to incorporate additional data, research, and suggestions from several hundred early users.

The TTM is a system dynamics simulation model [14, 15] that helps stewards negotiate tradeoffs as they navigate toward an equitable, thriving future. It is grounded in Elinor Ostrom’s Nobel Prize-winning work on shared stewardship of common resources [16, 17]. The current TTM closely matches the conceptual framework of the *Thriving Together Springboard* [8] and incorporates concepts such as targeted universalism [18], multisolving [19, 20], race-related stressors [21, 22], and the organizational capability trap [23], all of which have implications for investing in multiracial well-being. In general, the Thriving Together Model brings greater structure, evidence, and creativity to the tasks of negotiating investment priorities and playing out the likely consequences for multiracial well-being over time.

The TTM recognizes that all communities must contend with adversities: some are sudden like natural disasters and acts of violence; others last months or years like pandemics and economic recessions; and still others unfold over decades or longer like chronic diseases and structural racism. Everyone is adversely affected by these hardships, but certain groups are typically harmed more than others, especially members of marginalized racial/ethnic groups. The cumulative toll of these adverse forces ultimately affects one’s length of life (measured by life expectancy at birth) and life evaluation (measured by the Cantril Self-Anchoring Scale) [5]).

The revised TTM encompasses all of these topics. It equips stewards with a simplified but realistic representation of well-being dynamics in a multiracial population, including four main drivers of well-being at a community level, which in turn affect states of individual well-being (i.e., thriving %, suffering %) and life expectancy.

Investment priorities in the TTM involve allocating community assets (both funding and in-kind resources) among the following four drivers of well-being in a population:

- **Vital conditions:** Investments in this driver help to establish the vital conditions that everyone needs to experience health and well-being [9]. They include a thriving natural world, basic needs for health and safety, humane housing, meaningful work and wealth, lifelong learning, reliable transportation, as well as belonging and civic muscle, which is central to them all. In the TTM, belonging and civic muscle is represented separately because of its unique dynamic effects. The vital conditions framework renders essential concepts often described as “social determinants of health” in a form that is clear, concise, and built for concerted action across all sectors of society.
- **Belonging and civic muscle**: Investments in this driver enhance both belonging (i.e., feeling part of a community) and civic muscle (i.e., the power of people to work across differences) [4,11,24]. Belonging and civic muscle supports well-being directly, by enhancing social connection, and indirectly, by strengthening collective capacities (such as trusting relationships, power sharing, constructive nonviolence, and civic organizing) that, in turn, enlarge the pool of investable assets (both financial and in-kind) in a community.
- **Fairness in system design**: Investments in this driver help to assure just and fair treatment for all people as a matter of system design versus systemic exclusion across color, class, gender, ethnicity, and other lines that often divide population subgroups. Fairness in system design explains the pattern of opportunities and outcomes among people in a multiracial and multicultural society.
- **Urgent services capacity**: Investments in this driver help to meet the changing demand for services that alleviate life crises or urgent needs among those who are struggling and suffering [9]. They include acute care for illness or injury; addiction treatment (including overdose rescue and detoxification); crime response; environmental clean-up; homeless services; as well as unemployment benefits and food assistance. The TTM calculates the adequacy of urgent services over time by comparing urgent services capacity to changing levels of urgent need.

An analysis using an earlier version of the TTM explored how stewards in any given US community could respond to an unjust shock over the course of ten years [13]. Results suggested that an immediate emphasis on belonging and civic muscle coupled with fairness in system design could turbocharge virtuous dynamics and create an equitable path for renewal within a decade.

The revised model used here to study the ELTRR plan does not address short-term responses to a shock, but rather what it takes to move beyond an unacceptable status quo by embracing new priorities for well-being and justice. This can happen by strategically reallocating investment priorities among the four drivers, as in the original model, and by intentionally designing multisector actions to advance multiple goals at once, a practice known as “multisolving” [19, 20].

In addition, the revised model shows how thriving, suffering, and life expectancy vary by race/ethnicity [21, 22]. The updated model estimates the extent to which new priorities have the potential to generate not just more well-being overall, but also more equitable well-being in a community that begins with an entrenched pattern of multiracial inequity.

Our analysis of the ELTRR plan does not attempt to forecast exact outcomes in the future. Rather, it explores relative changes that could unfold over time in American communities if they were able—in cooperation with federal agencies—to move in the direction implied by the ELTRR. Because the ELTRR was designed to be a single unified plan, we regard all 68 programmatic recommendations as a cohesive package of new priorities.

The next section describes the revised model structure in greater detail, along with assumptions about initial conditions, and how we quantified changes to represent priorities described in the ELTRR.

### Model structure

**Fig 1:**
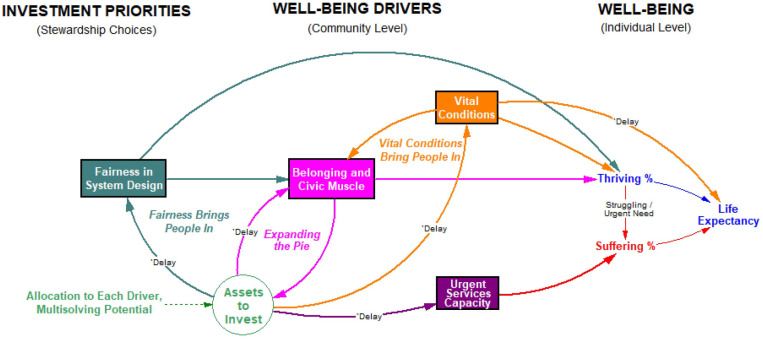
Overview of the Thriving Together Model.

Fig 1 shows the overall dynamic structure of the Thriving Together Model. Readers may also refer to the supplement for a complete list of equations and numerical assumptions, including uncertainty ranges for 11 coefficients and time constants. The supplement also contains results from sensitivity tests of single parameters, and from 5,000 random Monte Carlo simulations across all 11 uncertain parameters allowed to vary simultaneously.

Most of the dynamic structure remains the same as in the original model [13], including the four community-level drivers of well-being, each measured on a scale of 0–100%: vital conditions, belonging and civic muscle (BCM), fairness in system design, and urgent services capacity). Each driver is modeled in the aggregate, not broken out by subtypes. As before, vital conditions are the most important driver of individual thriving, with fairness and BCM also contributing. Suffering can be reduced to some degree when thriving improves (which reduces the amount of urgent need) and also through a greater adequacy of urgent services.

Average life expectancy is improved by greater thriving [25] and by more expansive vital conditions [26, 27], but it is reduced significantly by the extent of suffering [28]. The effect of vital conditions on life expectancy reflects all those mortality risk factors (like smoking, poor diet, physical inactivity, and slow-acting environmental pollutants) that are not immediately reflected in reduced well-being (struggling, suffering) and which show up, by and large, years later in the form of chronic diseases such as heart disease, diabetes, and cancer. More than 80% of lost life-years in the US are due to such noncommunicable diseases [29], with an average progression or latency period we have estimated at 15 years.

Also central to the model is the idea that the total pool of investable assets at any given time can expand in proportion to the community’s level of belonging and civic muscle. This ability to enlarge the asset pool is reflected in the reinforcing “expanding the pie” loop in Fig 1. The model also includes reinforcing feedback loops by which improvements in vital conditions or in fairness tend to boost BCM (and thus, the pool of investable assets) by bringing people in as active contributors to the community and drawing forth more resources through taxes, philanthropy, social entrepreneurship, volunteer efforts, and organized public work.

### Estimating well-being differentials by race/ethnicity

A significant new feature of the revised model is its breakout of thriving, suffering, and life expectancy by race/ethnicity, based on an analysis of historical data from 2008 to the present. To examine differentials, we focused primarily on data for 2016–2018 (a relatively recent pre-COVID period with large sample sizes in the survey data) to guide our estimates of starting conditions for the model. Table 1 presents a summary of these data along with a few other metrics that help to interpret the observed patterns of multiracial thriving and suffering.

**Table 1:** Selected US averages informing assumed differences in thriving, suffering, and life expectancy by race/ethnicity: Black; Hispanic; Asian; White; American Indian, Alaska Native.

			Race/Ethnicity				
Metric	Source	Data period	Black	Hispanic	Asian	White	AIAN*
**Thriving** %	Gallup [30]	2016-2018	52.4	56.4	61.6	56.0	n/a
Above poverty line %	Census [31]	2019	81.2	84.3	92.7	92.7	n/a
High school grad age 25-29 %	NCES [32]	2016-2019	91.7	83.7	97.0	95.7	88.2
**Suffering** %	Gallup [30]	2016-2018	3.3	3.2	1.9	3.7	n/a
Suicide death rate per 100k	KFF/CDC [33]	2020	7.7	7.5	6.8	16.8	23.9
Substance use disorder %	SAMHSA [34]	2015-2019	7.1	7.1	4.1	7.8	11.2
**Life expectancy years**	CDC [35]	2019	74.8	81.9	85.6	78.8	71.8
* AIAN = American Indian, Alaska Native							

Shaded cells indicate relative differences for each well-being metric, by race/ethnicity, ranging from dark green (best) to dark orange (worst).

Source: Multiple, as referenced

For thriving and suffering, we analyzed historical trends using data provided by the Gallup organization broken out for Black, Hispanic, Asian, and White subgroups. Unfortunately, Gallup did not report data for American Indian/Alaska Native or Native Hawaiian/Pacific Islanders [30].

In 2016–2018, **thriving** was lowest for Black Americans and highest for Asian, with Hispanic and White between the two. One might expect these differences in thriving to reflect similar differences in socioeconomic status. Table 1 shows that during the late 2010s, Black Americans were indeed much worse off than White and Asian on poverty [31], while Black and Hispanic groups were worse off than White and Asian on high school graduation [32].

In contrast, Black and Hispanic **suffering** was lower than White during the 2010s, and Asian suffering was the lowest of all. We found similar directional differences for two other metrics one might associate with suffering: suicide death rate [33] and the prevalence of substance use disorder [34]. In both cases, White Americans were worse off than Black, Hispanic, and Asian; only American Indian/Alaska Native people had higher rates than White on these measures.

For **life expectancy**, we analyzed annual data from the National Vital Statistics System on average life expectancy at birth broken out by race/ethnicity; 2019 is the first year that included data for Asian and for American Indian/Alaska Native [35]. Life expectancy for Black Americans was consistently four years less than that of White; Hispanic Americans were about three years greater than White; and Asian Americans were about six years greater than White. American Indian/Alaska Native people were lowest of all, about three years less than Black.

### Modeling future changes in well-being differentials

These historical differences provide a starting point for the model at Year 0, prior to any ELTRR implementation. Changes over time in life expectancy differentials between racial/ethnic groups are attributed in the model to changes in vital conditions and fairness. In particular, an improvement in vital conditions across the community is presumed to lead to smaller differences in suffering and life expectancy between racial/ethnic groups [27,36]; and an improvement in fairness is presumed to lead to smaller differences in thriving.

The idea that greater fairness in a community reduces racial/ethnic inequity in thriving was supported by statistical analysis. We had data from several national Gallup surveys for 2007–2021 on perceptions of how Black and Hispanic people are treated and their equality of opportunity [37]. Linear regressions revealed that the differences in thriving by race/ethnicity were strongly associated with a composite of three of the Gallup fairness measures: “agree Blacks treated fairly”, “agree Blacks have equal housing opportunity”, and “agree Black children have equal education opportunity”. We used this composite to configure the model’s initial estimate of fairness for the US at 54%. We estimated coefficients for the effects of fairness on thriving for all people of color in the model based on the regression results.

### Model initialization

We updated estimates for the initial values of overall thriving, suffering, and life expectancy (for all race/ethnic groups combined), as well as the four community-level well-being drivers (Table 2). In all cases, we used national US data from the late 2010s and early 2020s (as available) to initialize the model.

**Table 2:** Initial Values and Sources for Well-Being and Well-Being Drivers.

Variable	Initial Value	Source
**WELL-BEING** (Individual Level)		
**Thriving %**	53.5%	Data provided by Gallup
**Suffering %**	3.9%	Data provided by Gallup
**Life Expectancy at Birth**	78.9	[35]
**WELL-BEING DRIVERS** (Community Level; scale = 0–100%)		
**Vital Conditions**	80%	Composite of seven median measures across all US counties: people not in poverty, not in housing distress, having health insurance, doing some exercise, not smoking, graduating high school on time [38], and outdoor park within a half mile [39].
**Belonging and Civic Muscle**	50%	Composite of three measures, one for social and emotional support [40], one for voting in Congressional elections [41], and one for doing favors for neighbors [42]. All three of these measures were close to 50% during the late 2010s.
**Fairness in System Design**	54%	Composite of three Gallup fairness-to-Blacks measures: treated fairly, equal housing opportunity, equal child education opportunity [37].
**Adequacy of Urgent Services**	66%	Urgent Services Capacity estimated as 7.6%, the difference between urgent need of 11.5% and the initial suffering rate of 3.9%. The adequacy of urgent services is thus 7.6/11.5 = 66%. The urgent need estimate is based on the percentage of Americans who received benefits through the Supplemental Nutrition Assistance Program averaged across 2017–2021 [43].

Source: multiple, as indicated

### Modeling multisolving and gradual investment implementation

Another new feature of the revised model is a multisolving ratio, a measure of investment effectiveness that reflects the extent to which investments in one vital condition have additional benefits for other vital conditions as well. For example, an initiative to locate new affordable housing close to public transportation could help disadvantaged people fare better in at least two ways simultaneously, not just one. We represent such an initiative in the model as an increase in the multisolving ratio (see Supporting Information for calculation procedure).

A final new feature is the use of gradual linear ramps for the implementation of any new investment strategy. In the original model, with a time horizon of only 10 years, the emphasis was on quick implementation, acknowledging that shifting priorities might result in some unintended suffering in the first few years [13]. In the revised model, with a time horizon of 25 years, stewards can proceed more gradually at first to minimize unintended suffering. For this analysis, we have assumed a 5-year period for investment strategy ramp-ups.

### Estimating multisolving and asset allocation for the base run

The revised model is initialized in steady-state equilibrium, meaning that all output variables in the base run remain at their initial values. It is important to establish realistic values for all baseline parameters, so that alternative investment strategies (with new parameter assumptions) can be usefully compared. Informed by our fieldwork with stewards across the US, as well as by exploratory analysis of several ELTRR scenarios, we have tentatively settled upon a baseline value of 1.15 for the multisolving ratio, and a typical asset allocation split as follows: 50% to urgent services capacity, 30% to vital conditions, 10% to BCM, and 10% to fairness.

### Estimating multisolving and asset allocation under the ELTRR for model testing

We reviewed published descriptions of the 68 programmatic recommendations in the ELTRR [10] and for each one recorded: (a) its primary focus area, (b) any secondary focus areas listed, and (c) whether or not the description included an explicit focus on “equity”, “equitable”, or “fair” implementation. These terms are used frequently in the ELTRR document and describe an emphasis on expanding opportunity for racial/ethnic groups or other marginalized groups, similar to our concept “fairness in system design”, which more precisely differentiates a program’s means from its ends.

Table 3 summarizes the counts of the ELTRR’s recommended investment priorities, with one row for each of the seven primary focus areas and columns indicating the secondary focus areas along with fairness. For example, the first row indicates that there are 10 recommendations in the ELTRR that focus primarily on a Thriving Natural World, with all 10 of those also secondarily benefiting Basic Needs for Health and Safety, 5 benefiting Humane Housing, 1 benefiting Meaningful Work and Wealth, and so forth. The last row gives totals across all 68 recommendations, indicating a large number of secondary benefits: 103 for the first six vital conditions, 37 for belonging and civic muscle, and 38 for fairness.

**Table 3:** ELTRR recommendations category counts by primary and secondary areas.

		Secondary Focus Area Counts for Each Primary Area							
Primary Focus Area	Count	Thriving Natural World	Basic Needs for Health & Safety	Humane Housing	Meaningful Work & Wealth	Lifelong Learning	Reliable Transportation	Belonging & Civic Muscle	Fairness
**Thriving Natural World**	10		10	5	1	2	3	6	7
**Basic Needs for Health & Safety**	17	5		1	7	7	1	12	8
**Humane Housing**	9	3	2		2	1	2	2	4
**Meaningful Work & Wealth**	12	1	5	2		9	0	9	5
**Lifelong Learning**	6	0	5	0	4		1	3	4
**Reliable Transportation**	6	2	4	0	3	3		5	6
**Belonging and Civic Muscle**	8	1	2	1	2	5	1		4
**ALL PRIMARY AREAS**	**68**	**12**	**28**	**9**	**19**	**27**	**8**	**37**	**38**

Source: Authors’ analysis of the Federal Plan for Equitable Long-Term Recovery and Resilience [10]

We used the information for each of the 68 recommendations to estimate what the adoption of the full package could mean for equitable well-being in communities across America, represented here using national averages. We considered all recommendations together as a package, because that is how they were intended, not as a menu of independent initiatives. Accordingly, we developed an approach to estimate an overall multisolving ratio and an overall allocation of efforts across Vital Conditions, BCM, and fairness (see Supporting Information). The investment allocation to urgent services is considered at a later step because the ELTRR did not address urgent services.

Applying these assumptions to the package of 68 ELTRR recommendations produced an overall multisolving ratio of 1.30, as well as somewhat higher allocations to BCM and fairness.

To get the full four-way split of investment priorities required for the model, we also needed an assumption for the percentage of investment going to urgent services capacity. Table 4 presents two possibilities. One scenario (labeled “ELTRR_Urg50”) is to keep that percentage at 50% as it is in the base run. Another possibility (“ELTRR_Urg35”) is to reduce the urgent services allocation to 35% -- a relative reduction of 30%. A previous analysis of investment scenarios across 39 large urban counties [44] indicates that one could reasonably expect to reduce the need for urgent services by 29% after the first five years of enacting a well-designed portfolio of investments in vital conditions and BCM, with still more reduction after that. If one could reduce the asset allocation to urgent services by 30% over five years with little or no adverse effects, it would free up significant assets to apply the ELTRR approach more fully, perhaps leading to even greater well-being and longevity over time.

**Table 4:** Assumptions for three simulation runs. Changes from the base run in ELTRR_Urg50 and ELTRR_Urg35 are ramped up over five years starting in Year 1.

		Investment (Effort) Allocation, sum=100%			
**Run name**	**Multisolving** **ratio**	**Urgent Services Capacity**	**Vital Conditions**	**Belonging & Civic Muscle**	**Fairness**
**Base run**	1.15	50%	30%	10%	10%
**ELTRR_Urg50**	1.3	50%	27.3%	11.5%	11.2%
**ELTRR_Urg35**	1.3	35%	35.5%	15%	14.5%

Source: Authors’ analysis.

## Results

**Fig 2:**
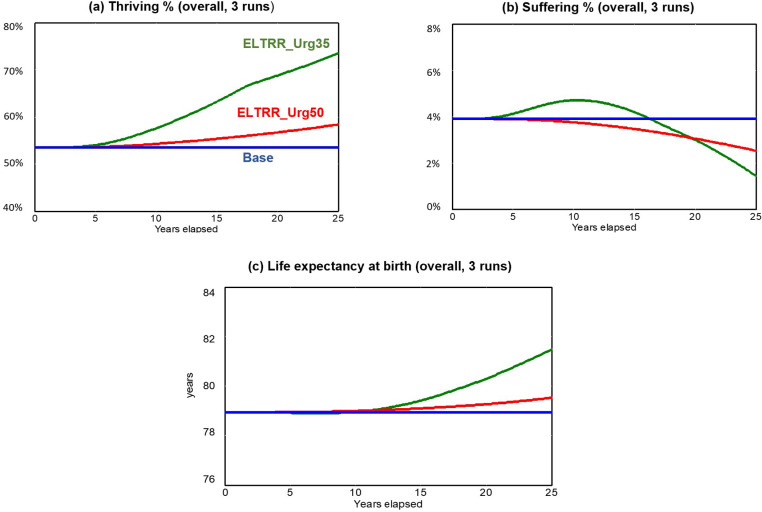
Well-being results over time (overall for all racial/ethnic groups) from three simulation runs. Blue line=base run, Red line=ELTRR_Urg50, Green line=ELTRR_Urg35.

**Fig 3:**
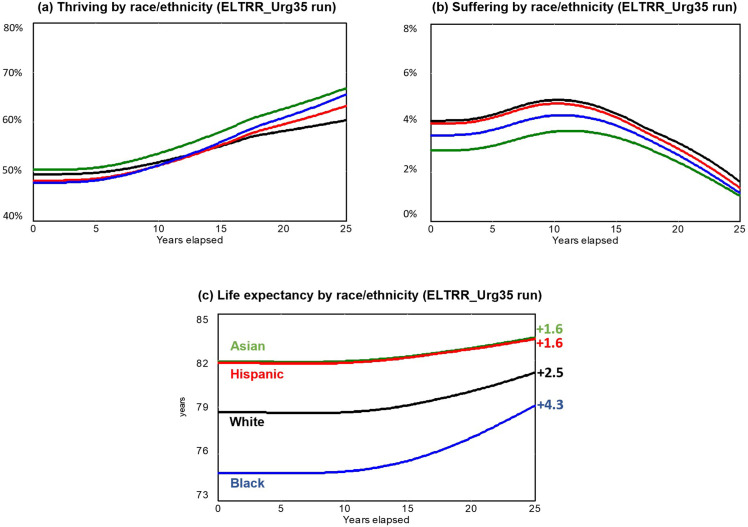
Well-being results over time by race/ethnicity from the ELTRR_Urg35 run. Blue line=Black, Red line=Hispanic, Green line=Asian, Black line=White. Source: Authors’ analysis.

Figs 2 and 3 compare each of the ELTRR scenarios described above as graphs over time; and Table 5 summarizes the main performance metrics in Year 25. Fig 2 compares each of the three ELTRR scenarios, showing overall trajectories for thriving, suffering, and life expectancy for all race/ethnic groups combined. Fig 3 concentrates on the best of those three scenarios (ELTRR_Urg35), showing separate trajectories for each race/ethnic group. Table 5 compares all three scenarios, showing the final results as of Year 25 for all four community-level well-being drivers, as well as for the individual-level measures of thriving, suffering, and life expectancy. Although these results reflect the model’s default numerical assumptions, the sensitivity tests reported in the supplement confirm that all of the following conclusions are robust across the identified uncertainty ranges.

**Table 5:** Key results from the three simulation runs as of Year 25.

	Result as of Year 25 by Run name			Percent Change
**Variable**	**Base run**	**ELTRR_Urg50**	**ELTRR_Urg35**	**ELTRR_Urg35** **vs. Base**
**WELL-BEING DRIVERS**				
**Vital conditions**	0.80	0.85	0.97	21%
**Belonging & civic muscle**	0.50	0.58	0.86	73%
**Fairness**	0.54	0.60	0.81	49%
**Adequacy of urgent services**	0.66	0.79	0.93	41%
**THRIVING %**				
Overall	0.535	0.584	0.735	37%
Black	0.516	0.580	0.782	52%
Hispanic	0.522	0.577	0.748	43%
Asian	0.555	0.615	0.800	44%
White	0.543	0.583	0.705	30%
**SUFFERING %**				
Overall	0.039	0.025	0.014	-63%
Max, Overall at any point	0.039	0.039	0.047	21%
Black	0.035	0.023	0.012	-67%
Hispanic	0.040	0.026	0.014	-66%
Asian	0.029	0.019	0.010	-64%
White	0.041	0.027	0.016	-61%
**LIFE EXPECTANCY AT BIRTH**				
Overall	78.9	79.5	81.5	3%
Black	74.8	75.6	79.1	6%
Hispanic	81.8	82.3	83.4	2%
Asian	81.9	82.3	83.5	2%
White	78.7	79.3	81.2	3%

Source: Authors’ analysis

The assumed changes in investment priorities for ELTRR_Urg50 and ELTRR_Urg35 (Table 4) ramp up over five years starting in Year 1, but the consequences unfold over many years, as the graphs show—a new steady state is still not reached even by Year 25. This gradual evolution reflects the persistent cycling of the model’s reinforcing loops, and especially the effect of belonging and civic muscle, which expands the pie of investable assets (shown in Fig 1).

By Year 25, the beneficial impacts are much greater under ELTRR_Urg35 than under ELTRR_Urg50, indicating the importance of the early shift from investing primarily in urgent services capacity toward a more balanced portfolio with the other three drivers (Vital Conditions, BCM, and Fairness). Table 5 shows that all four community-level well-being drivers (the first four rows) improve under ELTRR_Urg50, but they improve much more under ELTRR_Urg35. As a result, thriving, suffering, and life expectancy all show more robust improvements under ELTRR_Urg35 than under ELTRR_Urg50. This is true for all racial/ethnic groups combined and for each race/ethnicity separately.

In ELTRR_Urg35, thriving rises from its initial overall level of 53.5% to 73.5% (and to 70.5% or more for each race/ethnic group), and suffering declines from its initial overall level of 3.9% to 1.4% (and to 1.6% or less for each race/ethnic group). Average life expectancy rises by an average of 2.5 years, from 78.9 to nearly 81.5 years. It rises even more than that for Black Americans, by 4.3 years, 1.6 years for Hispanic and Asian, and 2.5 years for White. These differential gains reduce the Black-White life expectancy gap by 45% and are due to strong improvements in vital conditions and fairness in ELTRR_Urg35. (The fact that the Black-White gap is only closed by 45% by Year 25, and not more, is due to the 15-year average delay for chronic disease progression and latency noted above. Life expectancy disparities in Year 25 are thus a legacy of past unfairness—unfairness that improves substantially only after years of concerted effort. Even in an optimistic scenario like ELTRR_Urg35, the rapid rise in fairness occurs after Year 10.)

Why is the ELTRR_Urg35 scenario able to achieve so much more than ELTRR_Urg50? ELTRR_Urg50 achieves some success relative to the base run mostly because of the increase in the multisolving ratio, and also because of the greater allocation of investment to BCM, which starts to turn the “expand the pie” loop a bit faster. However, asset allocation to vital conditions is slightly less in ELTRR_Urg50 than the base run, which hinders the growth of thriving. This hindrance is removed in ELTRR_Urg35, which not only boosts the asset allocation to vital conditions but provides a much larger increase in the allocation to BCM. These changes allow the model’s reinforcing loops to turn much faster in ELTRR_Urg35 than they do in ELTRR_Urg50.

There is, of course, a concern that shifting some resources away from the investment in urgent services capacity, as in ELTRR_Urg35, might produce an unacceptable increase in the number of people suffering, at least in the short term. Indeed, Fig 2 does show a temporary rise in Suffering under ELTRR_Urg35, reaching a peak in Years 9–11 before it starts declining rapidly, crosses the ELTRR_Urg50 line, and drops below 3% by Year 20. But the peak is 4.7%, just 0.8 percentage points above the starting 3.9%, and within historical peaks, which at times have exceeded 5% (data provided by Gallup).

Why is the temporary rise in suffering in the ELTRR_Urg35 scenario relatively modest? One reason is that the ELTRR’s emphasis on multisolving produces somewhat greater thriving and lower suffering by Year 10 (see ELTRR_Urg50 results in Fig 2), thus helping to mitigate the short-term tradeoff that would otherwise have occurred due to the lower investment in urgent services capacity. A second reason is that the shift to less investment in urgent services capacity in ELTRR_Urg35 is phased in over five years rather than all at once, further helping to dilute any adverse impact.

## Discussion

This study began by asking: how can the United States make a great stride toward multiracial well-being? Our main finding defines at least one plausible path over the next 25 years in which the fraction of thriving people across the US could rise to 73.5% (up 20 percentage points from baseline), suffering could drop to 1.4% (down 2.5 percentage points), and average life expectancy could reach 81.5 years (up by 2.6 years). Moreover, those estimated gains arise from equitable progress across all racial/ethnic groups: every subgroup could improve, but the greatest gains would likely be among Black and Hispanic Americans, depending on the metric. Under this scenario, a more equitable, thriving future is possible if the US chooses to enact the investment priorities outlined in the federal interagency plan for Equitable Long-Term Recovery and Resilience (now known as *People and Places Thriving: The Federal Plan for Long-Term Resilience*) [12].

To analyze potential impacts from the ELTRR plan, we updated ReThink Health’s *Thriving Together Model*, incorporating new data and new features as described above. As far as we know, the revised model is the first dynamic model built to play out investment scenarios for community well-being and to report outcomes by race/ethnicity.

The baseline scenario used in this study resembles America’s real-world situation in the early 2020s: a diverse, multiracial population with stark differences in well-being among racial/ethnic subgroups and significant room to improve for everyone. For simplicity, all initial conditions are in dynamic equilibrium. Thus, the model starts with an entrenched status quo for well-being that sits far below America’s full potential – unless new investment priorities are enacted. Any projected changes in well-being and life expectancy under alternative scenarios are therefore directly attributable to assumed changes in nationwide investment priorities.

To underscore the realism of this status quo predicament, consider the following facts. America is infamous on the world stage for being unable to keep pace as other countries have increased life expectancy. “Between 1933 and 2021, 56 populous countries on multiple continents achieved higher life expectancy than the United States…[and] seventeen countries outperformed the United States for more than 50 years” [45]. US life expectancy also differs significantly by race/ethnicity [46]; and nearly half of US adults are either struggling or suffering, as opposed to thriving – a pattern that has remained roughly the same since nationwide tracking began more than 15 years ago [47]. To escape this troubling predicament, the ELTRR recommendations point the way toward a just transition for multiracial well-being.

### Plausibility of results

This model-based analysis is not a forecast but rather an exploration of what changes might be possible if federal agencies and communities across the US were able to move in the direction defined by the ELTRR. This study does not attempt to anticipate future events that could damage population well-being (such as economic recession, natural disasters, or violence). A prior paper explored the dynamics of responding to these sorts of severe shocks [13]. Instead, this study reveals why and how strategic investment priorities could influence multiracial well-being over time. Still, it is fair to ask whether the scenario-driven projections of a greatly improved future are plausible.

First, consider the projection of overall thriving, which climbs at the rate of 4–6 percentage points per five-year period from Year 5 to Year 25—an increase of 20 percentage points over 20 years. A study of thriving and suffering across all US counties [48], comparing the period 2008–2012 (midpoint 2010) to 2013–2017 (midpoint 2015) found that ten or more counties improved by 10–15 percentage points over this five-year period. We do not know how long such rapid improvement could continue, but these data show that the model’s rate of ascent is credible at least on a five-year basis.

Next, consider the model’s projection of overall suffering, which declines from about 4% initially down to 1.4% by Year 25. The same county-level study cited above [48] found that during the period 2013–2017, the four counties with the lowest percentage suffering were in the range of 0.4% to 0.7%. This result establishes the plausibility of a very low suffering percentage over at least a five-year period.

Finally, consider the model’s projection of life expectancy, which by Year 25 climbs to 81.5 years overall, and more than 83 years for the Asian and Hispanic subgroups. No state in the US has an overall life expectancy of more than 80 years [49], but there are individual counties with estimated life expectancies as high as 92 years [50]. Moreover, in 2019, many OECD countries had overall life expectancies of 82 years or greater, with Japan (84.4) and Switzerland (84.0) at the top [51].

### Significance

Although the *Thriving Together Model* remains a work-in-progress (with possible extensions discussed below), this exploratory analysis contributes in several ways to our understanding of collective well-being and the dynamics that drive potential improvement over time. Starting from such key concepts as shared stewardship of common resources [16, 17], targeted universalism [18], multisolving [19, 20], race-related stressors [21, 22], and the organizational capability trap [23], we built a quantified, testable simulation model that encompasses them all and closely matches the conceptual framework of the *Thriving Together Springboard* [6].

Through a series of systematic tests, including sensitivity analyses across multiple uncertain assumptions, we demonstrated the importance of three reinforcing feedback loops (shown in Fig 1) that each build Belonging and Civic Muscle and, in turn, “expand the pie” of investable assets in a community.

We also showed how investments to assure Fairness in System Design along with efforts to establish Vital Conditions are critical when seeking equitable gains among those racial/ethnic groups that are most marginalized and thus have the most to gain.

Moreover, we identified a highly impactful transition path, defined by the ELTRR_Urg35 scenario, in which the investment in urgent services capacity is gradually reduced, over five years, to make way for more investment in all other priorities. This scenario breaks the system out of a “capability trap” [23] (marked by an overreliance on urgent services) and incurs only a relatively small, temporary rise in suffering in the short-term on the way toward persistently better results afterwards. Finding that transition path helps us to see more generally that it may be possible to escape capability traps without confronting an unacceptable worse-before-better tradeoff.

The ELTRR_Urg35 scenario manages this by increasing the multisolving ratio and moving away gradually from a primary emphasis on urgent services. The moderately lower allocation to urgent services capacity (of 15 percentage points or 30% over five years) would not require dramatic closure of urgent care centers or immediate loss of safety net services. Instead, it could be accomplished by adjusting future allocations so they do not automatically replenish urgent service capacity in the same amount as before. Under this scenario, when urgent service contracts come up for renewal, approximately 6% per year could be sunsetted to achieve a 30% reduction over five years; and the sunset rate could be even lower if it were possible to deliver services more efficiently.

### Bringing the ELTRR scenario to life

Responding to the severity and unjust consequences of the COVID-19 pandemic, the ELTRR recommendations spell out unprecedented changes to routine business practices across scores of federal agencies – all oriented toward creating the vital conditions that everyone in the US needs to participate, prosper, and reach their full potential. The plan also commits to assure fair and just opportunities across all racial/ethnic groups, among other aspects of social equity. By design, the plan’s recommendations are within the power of each agency to enact immediately. They do not require any new legislative appropriations or new federal authorities. They only require the will and wherewithal of federal staff and partners across the country to see the potential for meaningful improvement in the lives of Americans and to bring these recommendations to life.

To make the ELTRR a reality, collective and harmonized interagency action needs to continue to expand across all parts of the federal government, as well as with state, local, territorial, and tribal governments and civil society partners. Leaders need to empower their staff to define outcomes of their efforts in terms of improvements in vital conditions and to seek collaborations that bear fruit for communities. For example, leaders of the Office of the Assistant Secretary for Health in the Department of Health and Human Services are both guiding development of initiatives and expanding strategic collaborations through an overt focus on “People and Places Thriving” (PPT) [12]. Officials from the other 46 agencies comprising the interagency PPT workgroup are making similar commitments. Together, they are introducing procedures to track progress, ensure active learning, and generate new evidence about the value of working through shared stewardship. The federal PPT plan is influencing numerous whole-of-government initiatives related to environmental justice, resilience, and well-being. Furthermore, state, tribal, territorial, and local leaders are beginning to craft their own complementary action plans seeking to make the most of federal connections and resources.

To realize the brighter future that this study reveals, stewards in every sector and every walk of life must move beyond the familiarity of conventional programs, policies, and investment priorities. Instead, they ought to devise a new generation of place-based and people-centered strategies that unlock the multisolving power of Belonging and Civic Muscle, Fairness, and Vital Conditions. Even now, innovators in communities across the country are beginning to craft community-led investment agendas with these features. See, for example, the recent report on, “Investing in Generational Change” produced through the award-winning work of BeWell Palm Beach County [52]. And they are not alone. Many other multisector stewardship groups are also beginning to negotiate regional well-being portfolios that fit their own aspirations, circumstances, and capacities [53, 54].

As more and more changemakers join a rising movement to thrive together, they may use the investment framework – and compelling results – from this study to inform their negotiations and commitments.

### Opportunities and extensions

The *Thriving Together Model* can be used by anyone who wants to explore what it takes to make a great stride toward multiracial well-being. Results reported here come from a model configured with nationwide data. However, stewards across the US may adjust the model’s starting conditions to better represent their own regions. That kind of regional configuration is possible with appropriate data and resources and has already begun with collaborating organizations representing states (e.g., Delaware) and multi-county regions (e.g., in southwestern Texas).

Although the model could, in theory, represent any number and type of population subgroups, this study reports results across four racial/ethnic groups. Other sociodemographic characteristics could be considered, such as age, educational attainment, or urban/rural residence. However, current data systems do not provide sufficiently detailed information to model multiple, overlapping characteristics. We chose to concentrate on multiracial well-being because it affirms an important, commonly understood idea of what it would mean to move toward an equitable future in which everyone has a fair chance to reach their full potential. Regrettably, we could only study four racial/ethnic groups because current data systems often exclude others, such as American Indian/Alaska Native and Native Hawaiian/Pacific Islander. These groups ought to be included in nationwide and regional data systems.

The ELTRR was designed to be a cohesive, interagency plan with 68 specific recommendations that we modeled as a single package. Future analyses could explore the potential contributions of smaller subsets of initiatives.

We may also consider disaggregating the Vital Conditions that we have so far modeled as a single combined index. Our Vital Conditions Index reflects an equally weighted average across six vital conditions and implicitly assumes that all six conditions move together over time and do not conflict with one another. That is a reasonable assumption supported by a prior disaggregated analysis [44]. Even so, there may be circumstances where it is worthwhile to track each vital condition separately.

Finally, future studies may examine not only the size of relative investments, but also how -- and by whom -- those investments are implemented. We have started to model and explore two such questions in particular:

1. ***What is the balance of lived/learned expertise?*** There is a growing consensus that investors ought to rely on a balanced mix of lived and learned expertise [55, 56]. An excessively top-down approach (relying only on “learned expertise”) may promise quicker results but tends to erode Belonging and Civic Muscle over time. Conversely, an exclusively grassroots or bottom-up approach (relying only on local “lived expertise”) may strengthen Belonging and Civic Muscle but could overlook successful policies and practices established in other places.
2. ***What is the threat of backlash and how strong is the commitment to avert it?*** US history is filled with instances where progress toward multiracial well-being – or even the suggestion of it -- provokes backlash [57, 58]. One may expect that moves toward greater fairness reflected in the ELTRR recommendations might similarly be undermined by resistance. Stewards using the model may want to open frank discussions about the threat of backlash in their regions and what it takes to build the will to thrive together [59].

## Supporting information

S1 Data Procedures for calculating multisolving ratio and level of effort; Results from sensitivity testing; and Equation list. (PDF)
