# Supplementary material for: How can the United States make a great stride toward multiracial well-being?
Source: PLoS One. 2025 Apr 16;20(4):e0319320. doi: 10.1371/journal.pone.0319320 (PMC12002441; doi:10.1371/journal.pone.0319320)
Supplement: S1 Data — (PDF) [file pone.0319320.s001.pdf]

How can the United States make a great stride toward multiracial well-being?

Simulating the potential of a federal interagency plan

Bobby Milstein, Jack Homer, Becky Payne, and Paul Reed

Supporting Information

PROCEDURES FOR CALCULATING MULTISOLVING RATIO AND LEVEL OF EFFORT

SENSITIVITY TESTING

Uncertainty Ranges (Table S1)

Sensitivity Tests for Single Parameters (Tables S2, S3, S4, S5)

Sensitivity Tests for Multiple Parameters (Figures S1, S2, S3, S4)

EQUATION LIST

PROCEDURES FOR CALCULATING THE MULTISOLVING RATIO AND LEVEL OF EFFORT

It was not practical to estimate all parameters uniquely for each of the 68 recommendations in the ELTRR, so instead we used the following uniform procedures to estimate the implications for the multisolving ratio and for how assets are allocated across the six vital conditions, BCM, and fairness.

Multisolving ratio

The multisolving ratio is a measure of investment effectiveness that reflects the extent to which investments in one of the six vital conditions have additional benefits for others of the six vital conditions as well. We represent such an initiative in the model as an increase in the multisolving ratio.

Assumptions for calculating the multisolving ratio:

1. Assume each vital condition's primary focus (N=60) contributes an impact value of 1.0 multiplied by the corresponding level of effort.
2. Assume each vital condition's secondary focus (N=103) contributes a relative impact value of 0.2.

Level of effort

Assumptions for calculating relative changes in the level of effort across drivers of well-being:

a. If the primary focus area is one of the six vital conditions, and the program also addresses BCM as a secondary priority, then BCM gets 30% of the total effort.

b. If the primary focus area is BCM, and the program also addresses any of the six vital conditions secondarily, then vital conditions gets 30% of the total effort.

Regardless of the focus area, if the program also addresses fairness, then fairness gets 40% of the total effort.

The investment allocation to urgent services is considered in a subsequent step, as described in the main narrative.

Example

An initiative to locate new affordable housing close to public transportation could help disadvantaged people fare better in at least two ways simultaneously, not just one. In the ELTRR, this is *Recommendation C* under Humane Housing, and it is described as having secondary benefits for Reliable Transportation as well as Fairness. Applying the rubric above, we would estimate that the total effort of this initiative is split 60% to vital conditions and 40% to fairness, and that the multisolving ratio for this initiative is 1.2.

Table S1. ELTRR sensitivity testing specifications, with worst-case settings (pink shading) and triangular distributions for Monte Carlo testing (N=5000 runs)

		Triangular distribution specifications				
**Uncertain parameter name**	**Default value**	Triangle Peak	Selection Min	Selection Max	Triangle Start	Triangle Stop
Invest impact delay time for VC	3 years	3	1	6	0	6
Invest impact delay time for BCM	3 years	3	1	6	0	6
Invest impact delay time for Fairness	3 years	3	1	6	0	6
Invest impact delay time for Urg Svcs	2 years	2	1	6	0	6
Exponent BCM building from Fairness	0.5	0.5	0.25	0.75	0	1
Exponent BCM building from VC	0.5	0.5	0.25	0.75	0	1
Exponent Thriving from VC	0.6	0.6	0.4	0.8	0.4	0.8
Exponent Thriving from Fairness	0.3	0.3	0.2	0.4	0.1	0.5
Exponent Thriving from BCM	0.15	0.15	0.1	0.2	0	0.3
Coefficient urgent need from non-thriving	0.5	0.5	0.25	0.75	0	1
Mortality risk manifestation time	15 years	15	12	20	10	20

FILE: TTM2o MC11.vsc

5300,M,1234,,0
Invest impact delay time for BCM=RANDOM_TRIANGULAR(1,6,0,3,6)
Invest impact delay time for Fairness=RANDOM_TRIANGULAR(1,6,0,3,6)
Invest impact delay time for VC=RANDOM_TRIANGULAR(1,6,0,3,6)
Invest impact delay time for USC=RANDOM_TRIANGULAR(1,6,0,2,6)
Exponent for BCM building from fairness=RANDOM_TRIANGULAR(.25,.75,0,.5,.1)
Exponent for BCM building from VC=RANDOM_TRIANGULAR(.25,.75,0,.5,1)
Exponent for thriving from VC=RANDOM_TRIANGULAR(.4,.8,.7,.9,1.1)
Exponent for thriving from fairness=RANDOM_TRIANGULAR(.3,.7,.2,.5,.8)
Exponent for thriving from BCM=RANDOM_TRIANGULAR(.1,.3,0,.2,.4)
Coefficient for urgent need from nonthriving=RANDOM_TRIANGULAR(.25,.75,0,.5,1)
Mortality risk manifestation time=RANDOM_TRIANGULAR(12,20,10,15,20)

Note: RANDOM_TRIANGULAR(selection min, selection max, triangle start, triangle peak, triangle stop)

Table S2. ELTRR single-parameter worst-case sensitivity testing: Community metrics at Year 25

Orange shading indicates worsening of 5+ percentage points (rounded) vs ELTRR_urg35 default.

		Result as of Year 25			
Run name	Parameter name and value	Vital conditions	BCM	Fairness	Adeq urg svcs
Baseline (no ELTRR)	No changes in uncertain parameters (all 11 at their default values)	0.80	0.50	0.54	0.66
ELTRR_urg50 (ELTRR but no pivot)		0.845	0.579	0.602	0.767
ELTRR_urg35 (default settings)		0.970	0.863	0.805	0.840
E_35 DtimeVC6	Invest impact delay time for Vital Cond = 6 years (default=3)	0.968	0.852	0.802	0.829
E_35 DtimeBCM6	Invest impact delay time for BCM = 6 years (default=3)	0.967	0.755	0.768	0.739
E_35 DtimeFair6	Invest impact delay time for Fairness = 6 years (default=3)	0.970	0.844	0.755	0.811
E_35 DtimeUSC6	Invest impact delay time for Urg Svcs = 6 years (default=2)	0.970	0.863	0.805	0.783
E_35 ExpoBCMFair25	Exponent BCM building from Fairness = 0.25 (default=0.5)	0.969	0.822	0.798	0.812
E_35 ExpoBCMVC25	Exponent BCM building from Vital Cond = 0.25 (default=0.5)	0.970	0.833	0.799	0.819
E_35 ExpoThrVC40	Exponent Thriving from Vital Cond = 0.4 (default=0.6)	0.970	0.863	0.805	0.809
E_35 ExpoThrFair20	Exponent Thriving from Fairness = 0.2 (default=0.3)	0.970	0.863	0.805	0.808
E_35 ExpoThrBCM10	Exponent Thriving from BCM = 0.1 (default=0.15)	0.970	0.863	0.805	0.818
E_35 CoeffUrgThr25	Coefficient for urgent need from non-thriving = 0.25 (default=0.5)	0.970	0.863	0.805	0.739
E_35 Morttime20	Mortality risk manifestation time = 20 years (default=15)	0.970	0.863	0.805	0.840

Comments:

* Three of the 11 sensitivity tests (*DtimeBCM6, DtimeUSC6, CoeffUrgThr25*) undermine the contribution of ELTRR_urg35 to Adeq Urg Svcsx. Also, *DtimeBCM6* undermines BCM; and *DtimeFair6* undermines Fairness.

Table S3. ELTRR single-parameter worst-case sensitivity testing: Thriving metrics at Year 25

Orange shading indicates worsening of 5+ percentage points (rounded) vs ELTRR_urg35 default.

Run name	Parameter name and value	Thriving result as of Year 25 All races	Black	Hispanic	Asian	White
Baseline (no ELTRR)	No changes in uncertain parameters (all 11 at their default values)	0.535	0.516	0.522	0.555	0.543
ELTRR_urg50 (ELTRR but no pivot)		0.584	0.580	0.577	0.615	0.583
ELTRR_urg35 (default settings)		0.735	0.782	0.748	0.800	0.705
E_35 DtimeVC6	Invest impact delay time for Vital Cond = 6 years (default=3)	0.732	0.778	0.745	0.799	0.702
E_35 DtimeBCM6	Invest impact delay time for BCM = 6 years (default=3)	0.709	0.747	0.719	0.770	0.684
E_35 DtimeFair6	Invest impact delay time for Fairness = 6 years (default=3)	0.718	0.753	0.727	0.777	0.696
E_35 DtimeUSC6	Invest impact delay time for Urg Svcs = 6 years (default=2)	0.735	0.782	0.748	0.800	0.705
E_35 ExpoBCMFair25	Exponent BCM building from Fairness = 0.25 (default=0.5)	0.727	0.772	0.740	0.793	0.698
E_35 ExpoBCMVC25	Exponent BCM building from Vital Cond = 0.25 (default=0.5)	0.729	0.775	0.742	0.796	0.700
E_35 ExpoThrVC40	Exponent Thriving from Vital Cond = 0.4 (default=0.6)	0.707	0.754	0.721	0.775	0.677
E_35 ExpoThrFair20	Exponent Thriving from Fairness = 0.2 (default=0.3)	0.706	0.753	0.720	0.774	0.676
E_35 ExpoThrBCM10	Exponent Thriving from BCM = 0.1 (default=0.15)	0.715	0.762	0.729	0.783	0.685
E_35 CoeffUrgThr25	Coefficient for urgent need from non-thriving = 0.25 (default=0.5)	0.735	0.782	0.748	0.800	0.705
E_35 Morttime20	Mortality risk manifestation time = 20 years (default=15)	0.735	0.782	0.748	0.800	0.705

Comments:

* None of the sensitivity tests significantly undermine the contribution of ELTRR_urg35 to Thriving.

Table S4. ELTRR single-parameter worst-case sensitivity testing: Suffering metrics at Year 25

Orange shading indicates worsening of 0.5+ percentage points (rounded) vs ELTRR_urg35 default.

		Suffering result as of Year 25					
**Run name**	**Parameter name and value**	**All races (year 25)**	**All races run Max**	**Black**	**Hispanic**	**Asian**	**White**
Baseline (no ELTRR)	No changes in uncertain parameters (all 11 at their default values)	0.039	0.039	0.035	0.040	0.029	0.041
ELTRR_urg50 (ELTRR but no pivot)		0.025	0.039	0.023	0.026	0.019	0.027
ELTRR_urg35 (default settings)		0.014	0.047	0.012	0.014	0.010	0.016
E_35 DtimeVC6	Invest impact delay time for Vital Cond = 6 years (default=3)	0.015	0.048	0.012	0.015	0.011	0.017
E_35 DtimeBCM6	Invest impact delay time for BCM = 6 years (default=3)	0.024	0.048	0.021	0.023	0.019	0.027
E_35 DtimeFair6	Invest impact delay time for Fairness = 6 years (default=3)	0.017	0.048	0.015	0.017	0.013	0.019
E_35 DtimeUSC6	Invest impact delay time for Urg Svcs = 6 years (default=2)	0.020	0.042	0.016	0.019	0.014	0.022
E_35 ExpoBCMFair25	Exponent BCM building from Fairness = 0.25 (default=0.5)	0.017	0.047	0.014	0.016	0.013	0.019
E_35 ExpoBCMVC25	Exponent BCM building from Vital Cond = 0.25 (default=0.5)	0.016	0.047	0.013	0.016	0.012	0.018
E_35 ExpoThrVC40	Exponent Thriving from Vital Cond = 0.4 (default=0.6)	0.018	0.048	0.015	0.017	0.013	0.020
E_35 ExpoThrFair20	Exponent Thriving from Fairness = 0.2 (default=0.3)	0.018	0.048	0.015	0.017	0.013	0.020
E_35 ExpoThrBCM10	Exponent Thriving from BCM = 0.1 (default=0.15)	0.017	0.047	0.014	0.016	0.012	0.019
E_35 CoeffUrgThr25	Coefficient for urgent need from non-thriving = 0.25 (default=0.5)	0.027	0.050	0.021	0.025	0.019	0.030
E_35 Morttime20	Mortality risk manifestation time = 20 years (default=15)	0.014	0.047	0.012	0.014	0.010	0.016

Comments:

* Three of the sensitivity tests (*DtimeBCM6, DtimeUSC6, CoeffUrgThr25*) undermine the contribution of ELTRR_urg35 to reduced Suffering (overall and by race). None of the tests causes a significant worsening of the Max Suffering bump.

Table S5. ELTRR single-parameter worst-case sensitivity testing: Average life expectancy at birth (ALEB) metrics at Year 25

Orange shading indicates worsening of 0.5+ year of life expectancy (rounded) vs ELTRR_urg35 default.

Run name	Parameter name and value	Life expectancy (ALEB) result as of Year 25				
		All races	Black	Hispanic	Asian	White
Baseline (no ELTRR)	No changes in uncertain parameters (all 11 at their default values)	78.9	74.8	81.8	81.9	78.7
ELTRR_urg50 (ELTRR but no pivot)		79.5	75.6	82.3	82.3	79.3
ELTRR_urg35 (default settings)		81.5	79.1	83.4	83.5	81.2
E_35 DtimeVC6	Invest impact delay time for Vital Cond = 6 years (default=3)	81.1	78.4	83.3	83.3	80.8
E_35 DtimeBCM6	Invest impact delay time for BCM = 6 years (default=3)	81.1	78.7	83.1	83.2	80.9
E_35 DtimeFair6	Invest impact delay time for Fairness = 6 years (default=3)	81.4	78.9	83.3	83.3	81.1
E_35 DtimeUSC6	Invest impact delay time for Urg Svcs = 6 years (default=2)	81.4	79.0	83.3	83.4	81.1
E_35 ExpoBCMFair25	Exponent BCM building from Fairness = 0.25 (default=0.5)	81.4	79.0	83.3	83.4	81.1
E_35 ExpoBCMVC25	Exponent BCM building from Vital Cond = 0.25 (default=0.5)	81.4	79.0	83.3	83.4	81.2
E_35 ExpoThrVC40	Exponent Thriving from Vital Cond = 0.4 (default=0.6)	81.3	79.0	83.2	83.3	81.1
E_35 ExpoThrFair20	Exponent Thriving from Fairness = 0.2 (default=0.3)	81.3	79.0	83.2	83.3	81.1
E_35 ExpoThrBCM10	Exponent Thriving from BCM = 0.1 (default=0.15)	81.4	79.0	83.3	83.4	81.1
E_35 CoeffUrgThr25	Coefficient for urgent need from non-thriving = 0.25 (default=0.5)	81.3	78.9	83.2	83.3	81.0
E_35 Morttime20	Mortality risk manifestation time = 20 years (default=15)	81.0	78.0	83.2	83.3	80.7

Comments:

* One of the sensitivity tests (*Morttime20*) undermines the contribution of ELTRR_urg35 to improved life expectancy (overall, and for Black and White). A second sensitivity test (*DtinmeVC6*) also undermines the contribution for Black specifically.

Figure S1. ELTRR Monte Carlo testing (11 uncertain parameters varied simultaneously, 5000 runs): Community metrics time graph envelopes
Key: central blue line=default parameter settings; Yellow band=25th to 75th percentile of outputs; Blue band=5th to 95th percentile of outputs; Grey band=all 5000 runs.

Comment: The 90% envelope (outer blue) shows relatively little sensitivity for VC, BCM, and Fairness. Adeq Urg Svcs shows a broader range of behavior, but always (for 90%) decreasing to Year 10-13 before increasing.

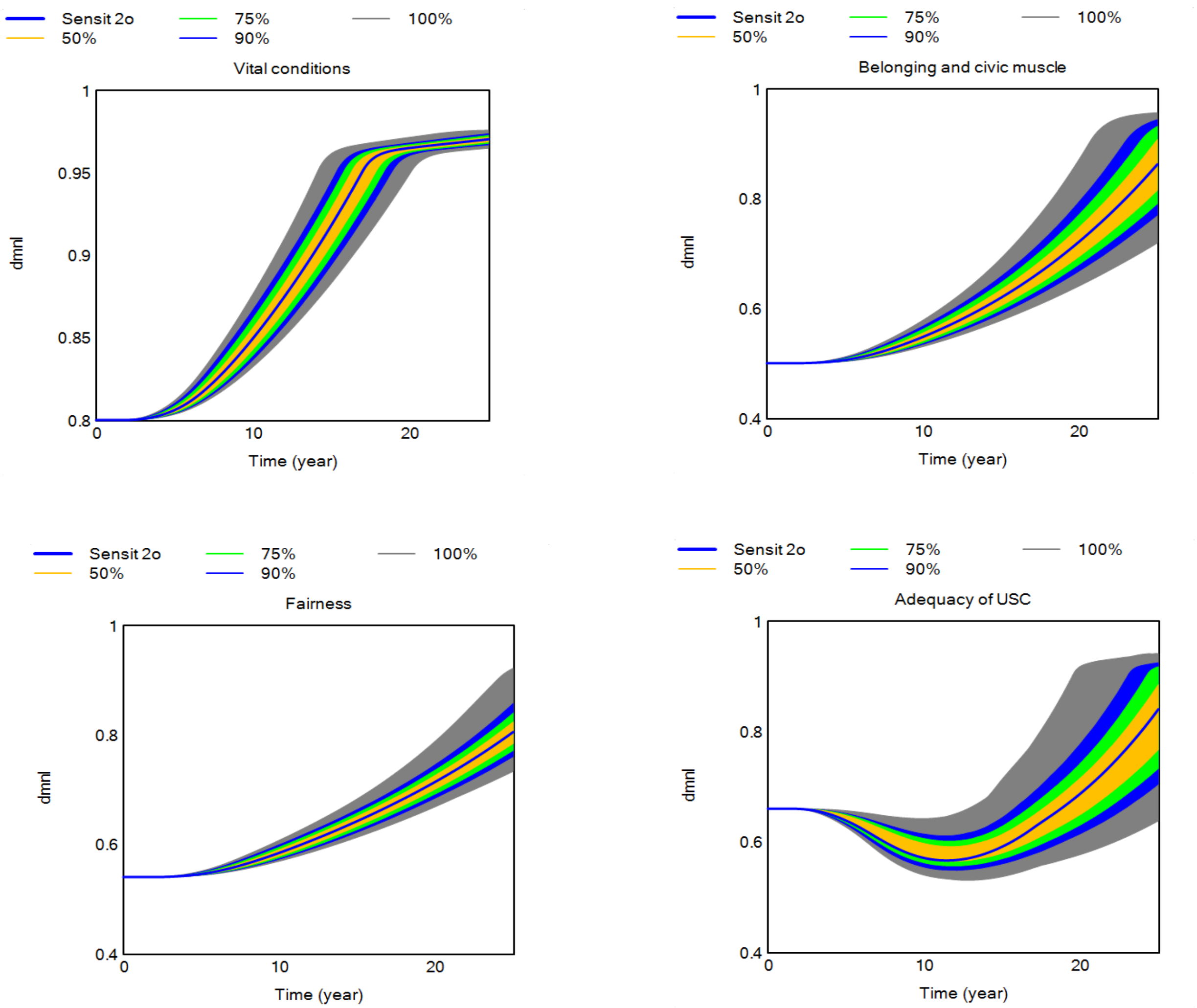

Figure S2. ELTRR Monte Carlo testing (11 uncertain parameters varied simultaneously, 5000 runs): Thriving time graph envelopes
Key: central blue line=default parameter settings; Yellow band=25th to 75th percentile of outputs; Blue band=5th to 95th percentile of outputs; Grey band=all 5000 runs.

Comment: The 90% envelope (outer blue) shows little variation in the growth pattern of Thriving (overall and by race/ethnicity).

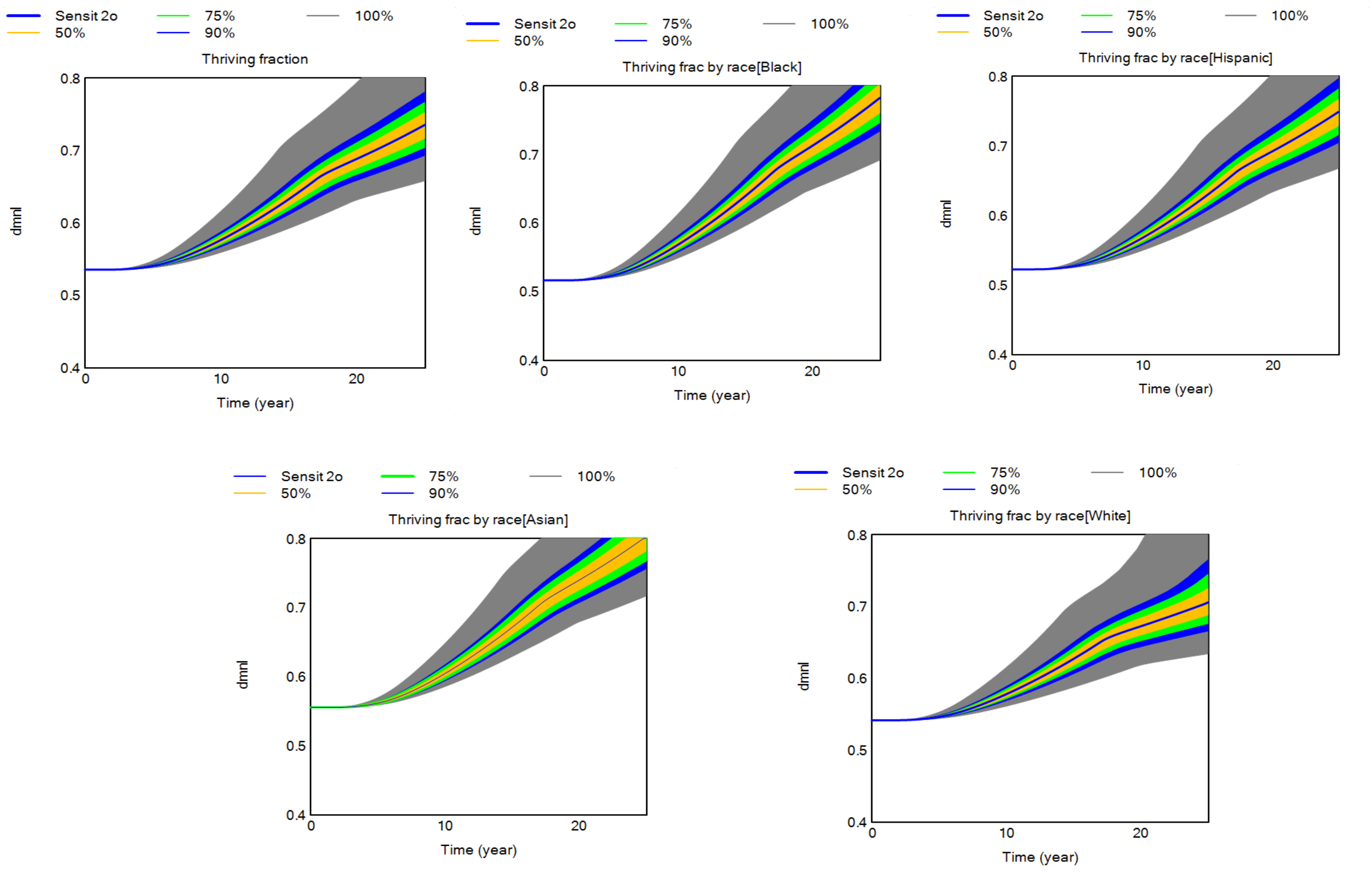

Figure S3. ELTRR Monte Carlo testing (11 uncertain parameters varied simultaneously, 5000 runs): Suffering time graph envelopes
Key: central blue line=default parameter settings; Yellow band=25th to 75th percentile of outputs; Blue band=5th to 95th percentile of outputs; Grey band=all 5000 runs.

Comment: The 90% envelope (outer blue) shows little variation in the downward pattern of Suffering (overall and by race/ethnicity).

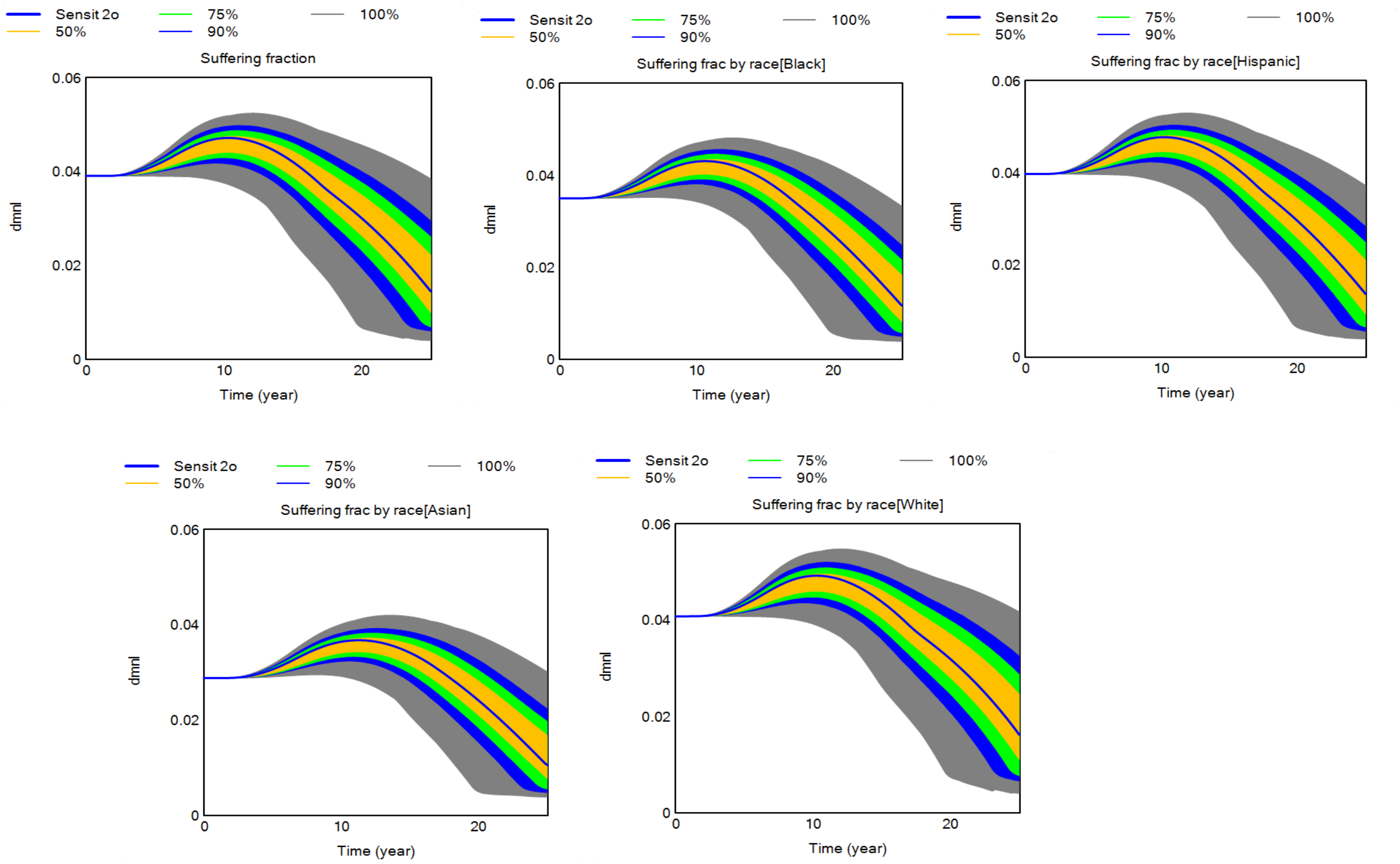

Figure S4. ELTRR Monte Carlo testing (11 uncertain parameters varied simultaneously, 5000 runs): Average life expectancy at birth (ALEB) time graph envelopes
Key: central blue line=default parameter settings; Yellow band=25th to 75th percentile of outputs; Blue band=5th to 95th percentile of outputs; Grey band=all 5000 runs.

Comment: The 90% envelope (outer blue) shows little variation in the growth pattern of life expectancy (overall and by race/ethnicity).

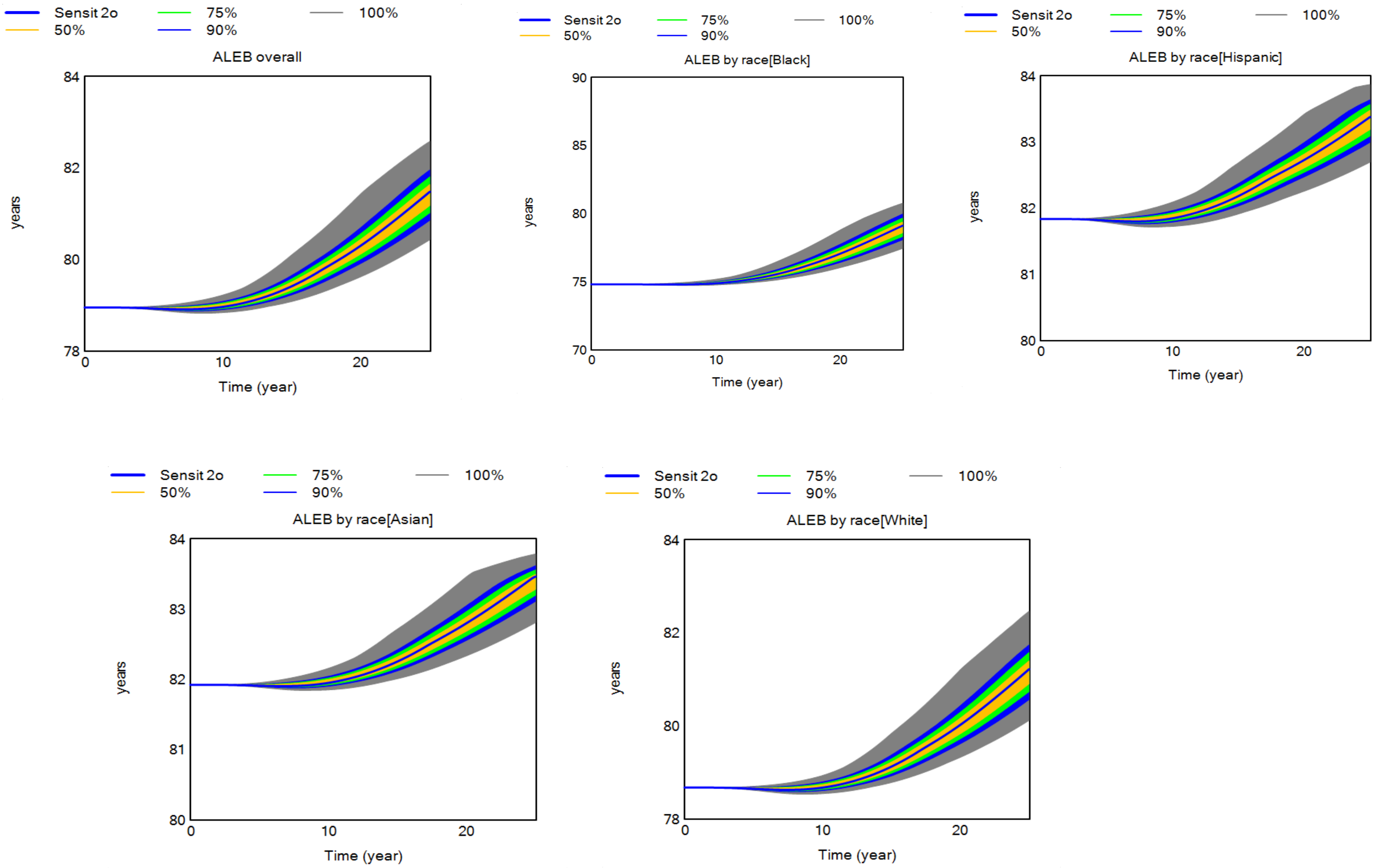

****
.Active
****

Active Equations

(001) Adequacy of USC= MIN(1, XIDZ(Urgent services capacity, MAX(0,Urgent need fraction-1e-05),1))
dimensionless *(USC: Urgent Services Capacity)*

(002) Adequacy of USC initial= 0.66
dimensionless

(003) Adequacy of USC saturation start point= 0.9
dimensionless

(004) ALEB by race[Race]= Indicated ALEB by race[Race] * ALEB overall/Indicated ALEB overall
years *(ALEB: Average Life Expectancy at Birth)*

(005) ALEB by race initial[Race]= INITIAL(ALEB by race[Race])
years

(006) ALEB of thriving by race[Race]= ALEB of thriving overall + ALEB of thriving delta by race[Race]
years

(007) ALEB of thriving delta by race[Race]= ALEB of thriving delta by race initial[Race] * (1-Reduction of mortality risks for the thriving)
years

(008) ALEB of thriving delta by race initial[Race]= -4.3,2.8,2.6,-0.4
years *(Race array: Black, Hispanic, Asian, White)*

(009) ALEB of thriving overall= ALEB of thriving overall initial + (ALEB of thriving overall max-ALEB of thriving overall initial)*Reduction of mortality risks for the thriving
years

(010) ALEB of thriving overall initial= 81
years

(011) ALEB of thriving overall max= 85
years

(012) ALEB overall= ALEB of thriving overall - AYLL from nonthriving
years

(013) ALEB overall initial= INITIAL(ALEB overall)
years

(014) ALEB ratio to avg by race[Race]= ALEB by race[Race]/ALEB overall
dimensionless

(015) AYLL from nonthriving= Struggling fraction*AYLL of struggling + Suffering fraction*AYLL of suffering
years *(AYLL: average years of life lost)*

(016) AYLL from nonthriving initial= INITIAL(AYLL from nonthriving)
years

(017) AYLL of struggling= 3
years

(018) AYLL of suffering= 20
years

(019) BCM building= BCM building no saturation * Effect of saturation on BCM building
1/year *(BCM: Belonging & Civic Muscle)*

(020) BCM building at investratio1= BCM building at investratio1 and initial fairness and VC * Effect of fairness on BCM building * Effect of VC on BCM building
1/year

(021) BCM building at investratio1 and initial fairness and VC= (BCM initial*BCM erosion rate initial)/(BCM initial*Invest allocation BCM initial)
1/year

(022) BCM building no saturation= DELAY1(BCM building at investratio1 * Invest index for BCM, Invest impact delay time for BCM)
1/year

(023) BCM erosion= Belonging and civic muscle * BCM erosion rate
1/year

(024) BCM erosion rate= BCM erosion rate at initial topdown*Effect of topdown on BCM erosion
1/year

(025) BCM erosion rate at initial topdown= BCM erosion rate initial + BCM erosion rate change*MAX(0, MIN(1, (Time-Erosion change ramp start time)/(Erosion change ramp end time-Erosion change ramp start time)))
1/year

(026) BCM erosion rate change= 0
1/year

(027) BCM erosion rate initial= 0.05
1/year

(028) BCM initial= 0.5
dimensionless

(029) BCM saturation start point= 0.9
dimensionless

(030) Belonging and civic muscle= INTEG (BCM building-BCM erosion, BCM initial)
dimensionless

(031) Change in ALEB= ALEB overall - ALEB overall initial
years

(032) Change in ALEB by race[Race]= ALEB by race[Race] - ALEB by race initial[Race]
years

(033) Change year for new invest decisions= 1
Year

(034) Coefficient for urgent need from nonthriving= 0.5
dimensionless

(035) Cumul avg change in ALEB= ZIDZ(Cumul change in ALEB, MAX(0,Time-Change year for new invest decisions))
years

(036) Cumul avg change in ALEB by race[Race]= ZIDZ(Cumul change in ALEB by race[Race], MAX(0,Time-Change year for new invest decisions))
years

(037) Cumul avg change in ALEB qualified= IF THEN ELSE(Max suffering frac<=Disqualifying max suffering frac, Cumul avg change in ALEB, MIN(0, Cumul avg change in ALEB))
years

(038) Cumul avg change in ALEB qualified by race[Race]= IF THEN ELSE(Max suffering frac<=Disqualifying max suffering frac, Cumul avg change in ALEB by race[Race], MIN(0, Cumul avg change in ALEB by race[Race]))
years

(039) Cumul change in ALEB= INTEG (Change in ALEB,0)
*years*years*

(040) Cumul change in ALEB by race[Race]= INTEG (Change in ALEB by race[Race],0)
*years*years*

(041) Disqualifying max suffering frac=0.05
dimensionless

(042) Effect of BCM on thriving= (Belonging and civic muscle/BCM initial)^Exponent for thriving from BCM
dimensionless

(043) Effect of fairness on BCM building= (Fairness/Fairness initial)^Exponent for BCM building from fairness
dimensionless

(044) Effect of fairness on thriving= (Fairness/Fairness initial)^Exponent for thriving from fairness
dimensionless

(045) Effect of nonthriving on urgent need= 1 + (Nonthriving ratio to initial-1)*Coefficient for urgent need from nonthriving
dimensionless

(046) Effect of saturation on BCM building= MIN(1, XIDZ(1-Belonging and civic muscle, 1-BCM saturation start point, 1))
1/year

(047) Effect of saturation on Fairness building= MIN(1, XIDZ(1-Fairness, 1-Fairness saturation start point, 1))
1/year

(048) Effect of saturation on USC building= MIN(1, XIDZ(1-Adequacy of USC, 1-Adequacy of USC saturation start point, 1))
1/year

(049) Effect of saturation on VC building= MIN(1, XIDZ(1-Vital conditions, 1-VC saturation start point, 1))
1/year

(050) Effect of topdown on BCM erosion= (1 + Slope for effect of topdown on BCM erosion*Invest topdown frac) / (1 + Slope for effect of topdown on BCM erosion*Invest topdown frac initial)
dimensionless

(051) Effect of topdown on invest impact= (1 + (Max effect of topdown on invest impact-1)*(Invest topdown frac^Exponent for effect of topdown on invest impact)) / (1 + (Max effect of topdown on invest impact-1)*(Invest topdown frac initial^Exponent for effect of topdown on invest impact))
dimensionless

(052) Effect of VC on BCM building= (Vital conditions/VC initial)^Exponent for BCM building from VC
dimensionless

(053) Effect of VC on thriving=(Vital conditions/VC initial)^Exponent for thriving from VC
dimensionless *(VC: Vital Conditions)*

(054) Erosion change ramp end time= 10
Year

(055) Erosion change ramp start time= 2
year

(056) Exponent for BCM building from fairness= 0.5
dimensionless

(057) Exponent for BCM building from VC= 0.5
dimensionless

(058) Exponent for effect of topdown on invest impact= 0.75
dimensionless

(059) Exponent for thriving from BCM= 0.2
dimensionless

(060) Exponent for thriving from fairness= 0.5
dimensionless

(061) Exponent for thriving from VC= 0.9
dimensionless

(062) Fairness= INTEG (Fairness building-Fairness erosion, Fairness initial)
dimensionless

(063) Fairness building= Fairness building no saturation * Effect of saturation on Fairness building
1/year

(064) Fairness building at investratio1= (Fairness initial*Fairness erosion rate initial)/(BCM initial*Invest allocation fairness initial)
1/year

(065) Fairness building no saturation= DELAY1(Fairness building at investratio1 * Invest index for fairness, Invest impact delay time for Fairness)
1/year

(066) Fairness erosion=Fairness * Fairness erosion rate
1/year

(067) Fairness erosion rate= Fairness erosion rate initial + Fairness erosion rate change*MAX(0, MIN(1, (Time-Erosion change ramp start time)/(Erosion change ramp end time-Erosion change ramp start time)))
1/year

(068) Fairness erosion rate change= 0
1/year

(069) Fairness erosion rate initial= 0.05
1/year

(070) Fairness initial=0.54
dimensionless

(071) Fairness saturation start point= 0.9
dimensionless

(072) Frac change to date in struggling= ZIDZ(Struggling fraction-Struggling fraction initial,Struggling fraction initial)
dimensionless

(073) Frac change to date in suffering= ZIDZ(Suffering fraction-Suffering fraction initial,Suffering fraction initial)
dimensionless

(074) Frac change to date in thriving= ZIDZ(Thriving fraction-Thriving fraction initial,Thriving fraction initial)
dimensionless

(075) Indicated ALEB by race[Race]= ALEB of thriving by race[Race] - AYLL of struggling*(1-Thriving frac by race[Race]-Suffering frac by race[Race]) - AYLL of suffering*Suffering frac by race[Race]
years

(076) Indicated ALEB overall= SUM(Indicated ALEB by race[Race!]*Popn frac by race initial[Race!])
years

(077) Invest allocation BCM= Invest allocation BCM initial + (Invest allocation BCM new-Invest allocation BCM initial) * MAX(0, MIN(1, (Time-Change year for new invest decisions)/Ramp duration to new invest allocation))
dimensionless

(078) Invest allocation BCM initial= 0.1
dimensionless

(079) Invest allocation BCM new= 0.1
dimensionless

(080) Invest allocation fairness= Invest allocation fairness initial + (Invest allocation fairness new-Invest allocation fairness initial) * MAX(0, MIN(1, (Time-Change year for new invest decisions)/Ramp duration to new invest allocation))
dimensionless

(081) Invest allocation fairness initial= 0.1
dimensionless

(082) Invest allocation fairness new= 0.1
dimensionless

(083) Invest allocation USC= Invest allocation USC initial + (Invest allocation USC new-Invest allocation USC initial) * MAX(0, MIN(1, (Time-Change year for new invest decisions)/Ramp duration to new invest allocation))
dimensionless

(084) Invest allocation USC initial= 1 - Invest allocation VC initial - Invest allocation BCM initial - Invest allocation fairness initial
dimensionless

(085) Invest allocation USC new= 1 - Invest allocation VC new - Invest allocation BCM new - Invest allocation fairness new
dimensionless

(086) Invest allocation VC= Invest allocation VC initial + (Invest allocation VC new-Invest allocation VC initial) * MAX(0, MIN(1, (Time-Change year for new invest decisions)/Ramp duration to new invest allocation))
dimensionless

(087) Invest allocation VC initial= 0.3
dimensionless

(088) Invest allocation VC new= 0.3
dimensionless

(089) Invest impact delay time for BCM= 3
years

(090) Invest impact delay time for Fairness= 3
years

(091) Invest impact delay time for USC= 2
years

(092) Invest impact delay time for VC= 3
years

(093) Invest index for BCM= Invest allocation BCM * Belonging and civic muscle * Effect of topdown on invest impact
dimensionless

(094) Invest index for fairness= Invest allocation fairness * Belonging and civic muscle * Effect of topdown on invest impact
dimensionless

(095) Invest index for USC= Invest allocation USC * Belonging and civic muscle * Effect of topdown on invest impact
dimensionless

(096) Invest index for VC= Invest allocation VC * Belonging and civic muscle * Effect of topdown on invest impact * VC multisolving ratio
dimensionless

(097) Invest topdown adjustment time= 2
years

(098) Invest topdown frac= DELAY1(Invest topdown frac decision, Invest topdown adjustment time)
dimensionless

(099) Invest topdown frac decision= Invest topdown frac initial + (Invest topdown frac new-Invest topdown frac initial) * MAX(0, MIN(1, (Time-Change year for new invest decisions)/Ramp duration to new invest allocation))
dimensionless

(100) Invest topdown frac initial= 0.5
dimensionless

(101) Invest topdown frac new= 0.5
dimensionless

(102) Lived learned experience frac= 1 - Invest topdown frac
dimensionless

(103) Max effect of topdown on invest impact= 1.3
dimensionless

(104) Max suffering frac= SAMPLE IF TRUE(Suffering fraction>Max suffering frac, Suffering fraction, Suffering fraction)
dimensionless

(105) Mortality risk manifestation time= 15
years

(106) Nonthriving ratio to initial= Struggling or suffering fraction/(1-Thriving fraction initial)
dimensionless

(107) Popn frac BIPOC series[Black]([(0,0)-(25,0.2)],(0,0.134),(10,0.138),(20,0.142),(25,0.144))
Popn frac BIPOC series[Hispanic]([(0,0)-(25,0.3)],(0,0.187),(10,0.211),(20,0.235),(25,0.246))
Popn frac BIPOC series[Asian]([(0,0)-(25,0.3)],(0,0.063),(10,0.071),(20,0.08),(25,0.083))
Dimensionless (BIPOC subset of Race array: Black, Hispanic, Asian)

(108) Popn frac by race[BIPOC]= Popn frac BIPOC series[BIPOC](Time)
Popn frac by race[White]= 1 - SUM(Popn frac BIPOC series[BIPOC!](Time))
dimensionless

(109) Popn frac by race initial[Race]= INITIAL(Popn frac by race[Race])
dimensionless

(110) Ramp duration to new invest allocation= 5
years

(111) Reduction of mortality risks for the thriving=DELAY3(ZIDZ(Vital conditions-VC initial,1-VC initial), Mortality risk manifestation time)
dimensionless

(112) Slope for effect of topdown on BCM erosion= 2
dimensionless

(113) Struggling fraction= Struggling or suffering fraction - Suffering fraction
dimensionless

(114) Struggling fraction initial= 1-Thriving fraction initial-Suffering fraction initial
dimensionless

(115) Struggling or suffering fraction= 1-Thriving fraction
dimensionless

(116) Suffering frac by race[BIPOC]= Suffering frac of nonthriving BIPOC[BIPOC] * (1-Thriving frac by race[BIPOC])
Suffering frac by race[White]= (Suffering fraction - SUM(Popn frac by race[BIPOC!]*Suffering frac by race[BIPOC!])) / Popn frac by race[White]
dimensionless

(117) Suffering frac of nonthriving= ZIDZ(Suffering fraction, Struggling or suffering fraction)
dimensionless

(118) Suffering frac of nonthriving BIPOC[BIPOC]= Suffering frac of nonthriving * Suffering ratio to avg BIPOC[BIPOC]
dimensionless

(119) Suffering fraction= Urgent need fraction * (1-Adequacy of USC)
dimensionless

(120) Suffering fraction initial= 0.039
dimensionless

(121) Suffering ratio to avg BIPOC[BIPOC]= 1 + (Suffering ratio to avg BIPOC initial[BIPOC]-1) * (1-VC gap closing frac vs initial)
dimensionless

(122) Suffering ratio to avg BIPOC initial[BIPOC]= 0.86,0.99,0.77
dimensionless

(123) Suffering ratio to avg by race[Race]= Suffering frac by race[Race]/Suffering fraction
dimensionless

(124) Thriving BIPOC pct gain per pctg point of Fairness gain[BIPOC]= 0.25,0.1,0.18
dimensionless

(125) Thriving delta BIPOC[BIPOC]= MIN(1-Thriving fraction, Thriving delta BIPOC initial[BIPOC] + (Fairness-Fairness initial)*Thriving BIPOC pct gain per pctg point of Fairness gain[BIPOC])
dimensionless

(126) Thriving delta BIPOC initial[BIPOC]= -0.019,-0.013,0.02
dimensionless

(127) Thriving frac by race[BIPOC]= MIN(Thriving fraction max, Thriving fraction + Thriving delta BIPOC[BIPOC])
Thriving frac by race[White]= MIN(Thriving fraction max, (Thriving fraction - SUM(Popn frac by race[BIPOC!]*Thriving frac by race[BIPOC!])) / Popn frac by race[White])
dimensionless

(128) Thriving fraction= MIN(Thriving fraction max, Thriving fraction initial * Effect of VC on thriving * Effect of fairness on thriving * Effect of BCM on thriving)
dimensionless

(129) Thriving fraction initial= 0.535
Dimensionless

(130) Thriving fraction max = 0.8
Dimensionless

(131) Thriving ratio to avg by race[Race]= Thriving frac by race[Race]/Thriving fraction
dimensionless

(132) Urgent need fraction= MIN(Urgent need fraction initial*Effect of nonthriving on urgent need, Struggling or suffering fraction)
dimensionless

(133) Urgent need fraction initial= Suffering fraction initial/(1-Adequacy of USC initial)
dimensionless

(134) Urgent services capacity= INTEG (USC building-USC erosion, USC initial)
dimensionless

(135) USC building= USC building no saturation * Effect of saturation on USC building
1/year

(136) USC building at investratio1= (USC initial*USC erosion rate initial)/(BCM initial*Invest allocation USC initial)
1/year

(137) USC building no saturation= DELAY1(USC building at investratio1 * Invest index for USC, Invest impact delay time for USC)
1/year

(138) USC erosion= Urgent services capacity * USC erosion rate
1/year

(139) USC erosion rate= USC erosion rate initial + USC erosion rate change*MAX(0, MIN(1, (Time-Erosion change ramp start time)/(Erosion change ramp end time-Erosion change ramp start time)))
1/year

(140) USC erosion rate change= 0
1/year

(141) USC erosion rate initial= 0.25
1/year

(142) USC initial= INITIAL(Urgent need fraction initial*Adequacy of USC initial)
dimensionless

(143) VC building= VC building no saturation * Effect of saturation on VC building
1/year

(144) VC building at investratio1= (VC initial*VC erosion rate initial)/(BCM initial*Invest allocation VC initial*VC multisolving ratio initial)
1/year

(145) VC building no saturation= DELAY1(VC building at investratio1 * Invest index for VC, Invest impact delay time for VC)
1/year

(146) VC erosion= Vital conditions * VC erosion rate
1/year

(147) VC erosion rate= VC erosion rate initial + VC erosion rate change*MAX(0, MIN(1, (Time-Erosion change ramp start time)/(Erosion change ramp end time-Erosion change ramp start time)))
1/year

(148) VC erosion rate change=0
1/year

(149) VC erosion rate initial= 0.05
1/year

(150) VC gap closing frac vs initial= (Vital conditions-VC initial)/(1-VC initial)
dimensionless

(151) VC initial= 0.8
dimensionless

(152) VC multisolving ratio= VC multisolving ratio initial + (VC multisolving ratio new-VC multisolving ratio initial) * MAX(0, MIN(1, (Time-Change year for new invest decisions)/Ramp duration to new invest allocation))
dimensionless

(153) VC multisolving ratio initial= 1.15
dimensionless

(154) VC multisolving ratio new= 1.15
dimensionless

(155) VC saturation start point= 0.95
dimensionless

(156) Vital conditions= INTEG (VC building-VC erosion, VC initial)
dimensionless

****
.Array
****
Subscripted Arrays

(157) BIPOC: Black, Hispanic, Asian

(158) Race: Black, Hispanic, Asian, White

****
.Control
****
Simulation Control Parameters

(159) FINAL TIME = 25
year

(160) INITIAL TIME = 0
year

(161) SAVEPER = TIME STEP
years

(162) TIME STEP = 0.125
years
